# An Evaluation of the Pathogenic Potential, and the Antimicrobial Resistance, of *Salmonella* Strains Isolated from Mussels

**DOI:** 10.3390/microorganisms10010126

**Published:** 2022-01-07

**Authors:** Antonio Lozano-León, Carlos García-Omil, Rafael R. Rodríguez-Souto, Alexandre Lamas, Alejandro Garrido-Maestu

**Affiliations:** 1 Laboratorio ASMECRUZ, Playa de Beluso s/n, 36939 Bueu, Spain; amcbeluso21@gmail.com (A.L.-L.); carlosgaromil@gmail.com (C.G.-O.); rafinol85@gmail.com (R.R.R.-S.); 2 Group CI8, Biomedical Research Center (CINBIO), Campus Universitario de Vigo, University of Vigo, 36310 Vigo, Spain; 3 Department of Analytical Chemistry, Nutrition and Bromatology, University of Santiago de Compostela, 27002 Lugo, Spain; alexandre.lamas@usc.es; 4 Food Quality and Safety Research Group, International Iberian Nanotechnology Laboratory, Av. Mestre José Veiga s/n, 4715-330 Braga, Portugal

**Keywords:** *Salmonella* spp., virulence genes, antibiotic resistance, mussel

## Abstract

*Salmonella* spp. and antimicrobial resistant microorganisms are two of the most important health issues worldwide. In the present study, strains naturally isolated from mussels harvested in Galicia (one of the main production areas in the world), were genetically characterized attending to the presence of virulence and antimicrobial resistance genes. Additionally, the antimicrobial profile was also determined phenotypically. Strains presenting several virulence genes were isolated but lacked all the antimicrobial resistance genes analyzed. The fact that some of these strains presented multidrug resistance, highlighted the possibility of bearing different genes than those analyzed, or resistance based on completely different mechanisms. The current study highlights the importance of constant surveillance in order to improve the safety of foods.

## 1. Introduction

*Salmonella* spp. is one of the most important foodborne pathogens worldwide, as highlighted by figures of salmonellosis in Europe, for which 52,702 confirmed cases were reported in 2020. In Spain, 3526 human salmonellosis were reported that year. At the distribution level, the highest number of *Salmonella*-positive samples was reported to be from meat products intended to be cooked (broiler, turkey and even pig and bovine meat) [1]. Regardless of these figures, *Salmonella* spp. is specifically regulated by the European Regulation 2073/2005 in a plethora of foodstuffs, including live mollusks [2]. In line with this Regulation, is the fact that the European Food Safety Authority (EFSA) reported that 2.01% of the outbreaks linked to the consumption of fish and fishery products were caused by *Salmonella* spp. [3]. In addition to this, another major health problem is the increased resistance of the microorganisms to antibiotics [4]. It was estimated that, every year, antimicrobial resistant microorganisms (ARMs) cause more than 23,000 deaths in the United States [5]. It has been previously reported that *Salmonella* spp. can be multidrug resistant (MDR) to antibiotics, thus becoming an even more important threat [6]. The EFSA data on antimicrobial resistance in 2018/2019, reported a 25.4% MDR in isolates of *Salmonella* spp. of human origin. Regarding the isolates from the food production chain, approximately 38% of the strains obtained from chicken, turkey and swine farms were MDR, but the highest number of MDR was found in pig carcasses, from which 43.3% of the *Salmonella* spp. recovered were MDR [7].

Spain is the third largest mussel producer worldwide, and Galicia accounts for 98% of the Spanish production [8]. In this region, the production has been developed in five different Rias: Vigo, Pontevedra, Arousa, Ares-Betanzos and Muros-Noia, but mainly in Arousa [9,10,11]. Previous studies have already reported the presence of pathogenic bacteria in this economically important mussel production area. These studies included pathogenic *Vibrio* and *Campylobacter* species, as well as Shiga toxin-producing *Escherichia coli*, thus highlighting the importance of screening for additional microorganisms, which can pose a risk for consumers [12,13,14]. In addition to these studies, *Salmonella* spp. was also detected and characterized in this area more than 10 years ago. All these facts highlight the importance of analyzing this food product [15,16]. For these reasons, the aim of the present study is to determine the presence of virulence genes in *Salmonella* spp. Strains, naturally isolated from mussels harvested in the Galician Rias (NW Spain), determine their antimicrobial resistance profiles, and compare these, if possible, with the results observed in previous studies conducted in the same geographical area and in other parts of the world.

## 2. Materials and Methods

### 2.1. Sampling

**Figure 1:**
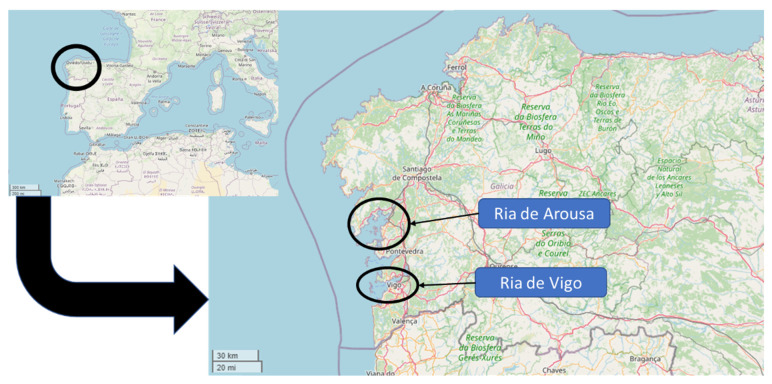
Geographical area for mussel sample collection.

Mussel samples, harvested in the Rias of Arousa and Vigo (Figure 1), were analyzed from 2012 to 2016. The samples were collected, as previously reported [12]. Briefly, the mussels were collected from the rafts, placed in sterile bags and transported under refrigeration to the laboratory. Upon arrival, they were washed with tap water, opened and collected under aseptic conditions (dead or broken mussels were discarded). Twenty-five grams of mussel (tissue and liquor), obtained from a minimum of 15 individuals, were weighted and diluted 1/10 in 225 mL of buffered peptone water (BPW, BioMérieux, S.A., Marcy l’Etoile, France), homogenized for 90 s and incubated at 37 °C for 18 ± 2 h. Once the incubation was completed, the DNA was extracted and analyzed by qPCR, as described below.

### 2.2. DNA Extraction Protocol

DNA extraction was performed with the PrepSEQ™ Rapid Spin Sample Preparation Kit (Applied Biosystems, Foster City, CA, USA) from enrichment broths, following the manufacturer’s instructions. Briefly, 750 µL was loaded in a sterile tube and centrifuged for 3 min at 14,000 rpm. The supernatant was discarded and the pellet was resuspended in sterile distilled water and loaded in a spin column previously placed in a sterile tube and centrifuged again in the same conditions. The column was discarded as well as the supernatant. The pellet was resuspended in 50 µL of Lysis Buffer plus 5 µL of proteinase K solution (Sigma-Aldrich, St. Louis, MO, USA), and homogenized by vortexing. Then, the samples were incubated at 95 °C for 15 min. After incubation, the samples were left at room temperature for 3 min and were centrifuged for 1 min at 14,000 rpm. Finally, 250 µL of milli-Q water was added and the samples were centrifuged for 1 min at 14,000 rpm. All extracts were stored at −80 °C until qPCR analysis.

### 2.3. Screening of Salmonella spp. by qPCR in Mussel Samples

The detection of *Salmonella* spp. was performed with the MicroSEQ^®^ (Applied Biosystems, Foster City, CA, USA) kit following the supplier’s instructions. The DNA extraction from the pre-enriched cultures was performed, as described in M & M 2.3, and 5 µL of DNA was used as a template. The reactions were performed in a final reaction volume of 20 µL, and run in a 7500 Fast Real Time PCR System Thermal Cycler (Applied Biosystems, Foster City, CA, USA) with the following thermal profile: 95 °C for 5 min (Hot-Start) followed by 40 cycles of denaturation at 95 °C for 5 s and annealing–extension at 60 °C for 30 s. Positive qPCR results were confirmed streaking the pre-enrichment culture on CHROMID^®^ *Salmonella* ELITE agar, and incubated at 37 °C for 18 ± 2 h. Typical colonies were subcultured on triptic soy agar (TSA), incubated at 37 °C for 18 ± 2 h and then biochemically identified with API20E (BioMérieux, S.A., Marcy l’Etoile, France).

### 2.4. Genetic Characterization of the Isolates

#### 2.4.1. Screening for Virulence Genes

A total of five virulence genes were screened (Table 1). These were: *invA* (first gene in an operon which is thought to trigger the internalization of *Salmonella* spp.); *hilA* (important in the regulation of the Type III secretion system); *sopB* (encodes for *Salmonella* outer protein B, part of the Type III secretion system); *pefA* (plasmid encoded fimbriae) and *spvC*, encoded in a virulence plasmid, whose expression helps the replication of *Salmonella* in the host cell reticuloendothelial system, especially in cells of the liver and spleen [17]. The detection of these genes was performed, as previously described [18,19,20,21,22].

#### 2.4.2. Screening for Antibiotic Resistance Genes

In addition to these virulence factors, genes implicated in the resistance to aminoglusides and β-lactams were also screened. The selected genes were *armA*, *rmtA*, *rmtB*, *rmtC*, *rmtD* and *npmA* for the aminoglucosides, while *bla*_CTX-M-15_ was targeted to assess resistance to β-lactams. The detection of all these genes was performed, as previously described [23,24]. A detailed list of the primers used is provided in Table 1. Additionally, the specific thermal profiles for each genetic target is provided in Table 2. All primers were purchased from Sigma-Aldrich (St. Louis, MO, USA).

### 2.5. PCR Tests for Isolate Characterization

The detection of the different virulence and antimicrobial resistance genes was performed by PCR in a final reaction volume of 25 µL, with a final primer concentration of 1000 nM and 2 µL of template. To this end, a PTC-200 (MJ Research, Waltham, MA, USA) thermocycler was used, programed with the specific thermal profile detailed in Table 2.

After amplification, the fragments were visualized after electrophoresis in a 1.8 % agarose gel (Promega, Madison, WI, USA) prepared with 1 X Tris-Borate buffer (TBE 1 X, Tris-base 89 mM, boric acid 89 mM, 2 mM EDTA, pH 8.3), to which 5 µL of a 1 % ethidium bromide solution (PanReac-AppliChem, Barcelona, Spain) was added. A total of 5 µL of the PCR product was mixed with another 5 µL of acridine orange loading buffer (PanReac-AppliChem, Barcelona, Spain), and fully loaded in the gel. Additionally, 2 µL of 100 bp DNA ladder (Nippon Genetics Europe, Düren, Germany) was mixed with 8 µL of loading buffer. The electrophoresis was performed in a kuroGel Midi Plus 15 (VWR, Radnor, PA, USA) cuvette with a Model 300 V programable power source (VWR, Radnor, PA, USA), and the fragments were migrated for 1 h and 30 min at 60 V after what was visualized under UV light with a Benchtop UV transilluminator (UVP, Upland, CA, USA).

### 2.6. Antimicrobial Resistance Test

The antimicrobial resistance tests were performed on a VITEK^®^2 (BioMérieux, S.A., Marcy l’Etoile, France) using the AST-N244 cards (cefalotin; cefditoren; nitrofurantoin; tobramycin; fosfomycin; ampicillin; amoxicillin/clavulanic acid; piperacillin/tazobactam; cefuroxime; cefuroxime/axetil; cefoxitin; cefotaxim; ceftazidime; cefepime; ertapenem; imipenem; amikacin; gentamicin; nalidixic acid; ciprofloxacin; tigecycline; and trimethoprim/sulfamethoxazole). The classification as sensitive (S), intermediate (I) or resistant (R) was based on the criteria determined by the “Clinical and Laboratory Standards Institute” [25].

## 3. Results and Discussion

### 3.1. Prevalence and Serotypes of Salmonella enterica

Over the period of study, a total of 27 *Salmonella* spp. isolates were recovered from the mussel samples. A detailed list of the strains isolated, along with the sampling site, is provided in Table 3. Martinez et al. previously evaluated the presence of foodborne pathogens by PCR, in bivalve mollusks from the Ria de Arousa, over a period of 18 months [26]. In the mentioned study, 22 mussel sample batches were negative for *invA*, thus negative for *Salmonella* spp. The low prevalence of *Salmonella* in this type of product, along with the small number of batches tested, can justify these results. In line with this observation, Lozano-Leon et al. reported 19 *Salmonella* positive samples from 5907 mussel batches, representing a prevalence of 0.3% [27]. This prevalence contrasts with that observed in other countries where, for instance, Zahli et al. found a prevalence of *Salmonella* of 19.15% in mussels collected from Moroccan markets; likewise, Mannas et al. and Setti et al. found a *Salmonella* prevalence of 15.4% and 10% respectively, in mussel samples collected from the Atlantic coastline of Morocco [28,29,30]. Although an attempt was made to establish a correlation between the environmental factors, such as rainfall and fecal contamination with the prevalence of *Salmonella*, the results were contradictory [29,30].

*S. typhimurium* was the most prevalent serotype (4/27), followed by *S. rissen* (3/27) and *S. senftenberg* (3/27). The information available about the serotypes present in mussel samples is limited, but the presence of *S. senftenberg* in mussels has to be pointed out as this serotype was associated with the persistent contamination in high saline environments in mussel facilities, between 1998 and 2002 [31]. Curiously, Setti et al. detected the presence of this serotype in the seawater and sediment of Morocco’s Atlantic coast, but not in mussels [29]. Previous studies carried out in the Rias of Vigo, Pontevedra, Arousa and Muros-Noia, between 1998 and 2002, in mussels, seawater and mollusk depuration plants, found that *S. senftenberg*t was the most prevalent *Salmonella* serotype [15,32]. In addition to this, the isolates of this serotype were higher in the Ria de Arousa in comparison to the other Rias. In the present study, all the isolates of *S. senftenberg* were from Ria de Arousa. The data from both previous studies mentioned, and the fact that this serotype is still isolated in mussel samples from Ria de Arousa, seem to indicate that this serotype can be endemic to this area. The *S. senftenberg* isolates characterized by Martinez-Urtaza et al., presented a rugose morphotype, which is associated with a high capacity to produce biofilm [31,33]. This can explain the persistence of these strains in mussel production plants, and that they are a permanent source of contamination in Ria de Arousa. Likewise, the fact that this serotype is isolated in marine samples from other parts of the world, seems to indicate a high adaptation of this serotype to high salt concentrations.

*S. typhimurim* was also one of the most prevalent serotypes isolated from the Galician marine environments, in previous studies conducted by Martinez-Urtaza et al. [15,16]. The presence of this serotype in mussels, represents an important concern for public health since, together with *S. enteritidis*, it is one of the most pathogenic serotypes of *Salmonella*. Factors, such as densely populated areas and inefficient wastewater treatments, can be related to the prevalence of *Salmonella* in the marine environment. In line with this hypothesis, a study carried out on the coast of Colombia established a relationship between inefficient wastewater treatment systems and the presence of *Salmonella* in beach waters [34]. *S. typhimurium* is the second serotype most commonly associate with human salmonellosis in the European Union, and therefore highly populated coastal regions can suffer a potential contamination with human pathogenic *Salmonella* serotypes [35]. On the other hand, Galicia is an important farming region with a large number of cattle, swine and poultry farms. These farms can contaminate the nearby aquifers and rivers with bacterial pathogens that can end up in the sea. It is worth noting that the serotype Senftenberg was also isolated from Galician poultry farms, between 2011–2015 [36]. Finally, it is also important to mention that *S. agona* was isolated in this study, and also showed a high prevalence of molluscan shellfish by Martinez-Urtaza et al. [15]. Isolates belonging to this serotype have been characterized by their high capacity to form biofilms, which can partly explain why this serotype can be a permanent source of contamination [37].

### 3.2. Genetic Characterization of the Isolates

#### 3.2.1. Screening for Virulence Genes

**Figure 2:**
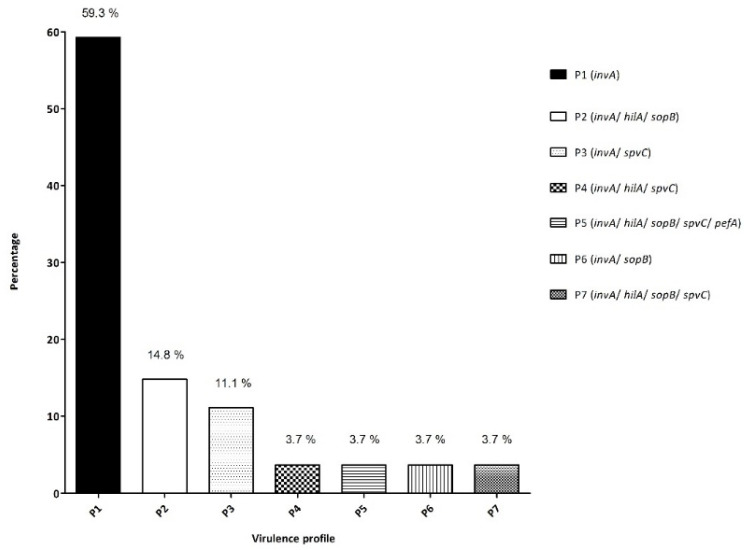
Virulence profiles obtained for the 27 isolates analyzed, based on the presence of the genes *invA*, *hilA sopB*, *pefA* and *spvC*.

All the strains isolated were positive for *invA* (100%); 7 were positive for *hilA* and *sopB* (25.9%), 6 for *spvC* (22.2%) and only 1 was positive for *pefA* (3.7%). The strains analyzed can be classified into 7 different groups, attending to the virulence genes presented, which would be: P1 (*invA*); P2 (*invA*/*hilA*/*sopB*); P3 (*invA*/*spvC*); P4 (*invA*/*hilA*/*spvC*); P5 (*invA*/*hilA*/*sopB*/*spvC*/*pefA*); P6 (*invA*/*sopB*) and P7 (*invA*/*hilA*/*sopB*/*spvC*), as depicted in Figure 2 and summarized in Table 4, and only one isolated (*S. typhimurium* 268) presented all the virulence genes analyzed. It is not surprising that all the strains were positive for *invA*, as this gene has been extensively used for the specific detection of *Salmonella* spp. by a wide variety of DNA amplification methods, being also selected for interlaboratory validation studies [38,39,40]. However, in the present study, only 25.9% of the isolates were positive for the main virulence transcriptional regulator of *Samonella* Pathogenicity Island 1 (SPI-1) *hilA*, and 59.3 % of isolates did not present any gene other than *invA* [41]. A study previously conducted by Campioni et al., reported higher percentages of *sopB* and *spvC*. It is noteworthy that the study focused on *spvB,* but these two genes belong to the same operon, *spv*RABCD [42]. These differences can be explained either by a difference in the pathogenic potential of the strains, or directly by the fact that these genes can be conserved in certain serovars (Campioni’s study was restricted to *Salmonella enteritidis*). In this sense, Lamas et al. observed that *spvC* was present in only 44.8% of the *Salmonella* strains isolated from Galician poultry farms. However, that gene was mainly present in *S. typhimurium* and *S. enterica* subsp *arizonae*, in which this gene is chromosomally encoded, but almost absent in the other serotypes of *S. enterica* subsp. *enterica* [43]. Ammar et al., like Campioni, analyzed the strains related with human disease, and obtained high percentages of *hilA* (88%), *sopB* (41.2%) and *pefA* (41.2%); the only discrepancy among them was for *spv*C, as only 5.9% of Ammar’s strains possessed the gene [44]. The results obtained in the present work are aligned with those reported by Gharieb et al., who indicated the percentages of 10%, 30 % and 16.7–30% for *pefA*, *hilA* and *sopB* respectively [45].

Attending to the geographical distribution, it is worth commenting that 25.9% of the isolates that presented more than one virulence gene belonged to the Ria of Arousa, while 14.8% belonged to the Ria of Vigo. Of particular importance is the fact that P7 (all 5 virulence genes) was obtained from a sample harvested in Vigo, and P5 (4 virulence genes) belonged to a sample harvested in Arousa. Caution must be taken when extracting conclusions related to the abundance as, due to the fact that certain areas are more productive, more samples were analyzed. In line with this, in previous studies, a bias existed towards the Ria of Arousa, due to a higher number of samples harvested and analyzed from this area, as a consequence of having a higher production [12,13]. The current study adds to the others previously published, informing the presence of different pathogens in this economically important area, and highlighting the importance of depuration for the safety of the consumers.

#### 3.2.2. Screening for Antimicrobial Resistance Genes

Regarding the presence of antimicrobial resistance genes, none of the seven genetic targets selected were detected in any of the isolates obtained. The fact that none of the genes screened were positive, but the isolates presented phenotypic resistance (see below) to many aminoglycosides, suggests the presence of additional mechanisms of resistance to these compounds, or the presence of other genes rather than those screened, such as *bla*_SHV_, *bla*_TEM_ or other *bla*, instead of M-15 in ESBL strains or *aacC* for the resistance of aminoglycosides [46,47,48].

### 3.3. Antimicrobial Resistance

**Figure 3:**
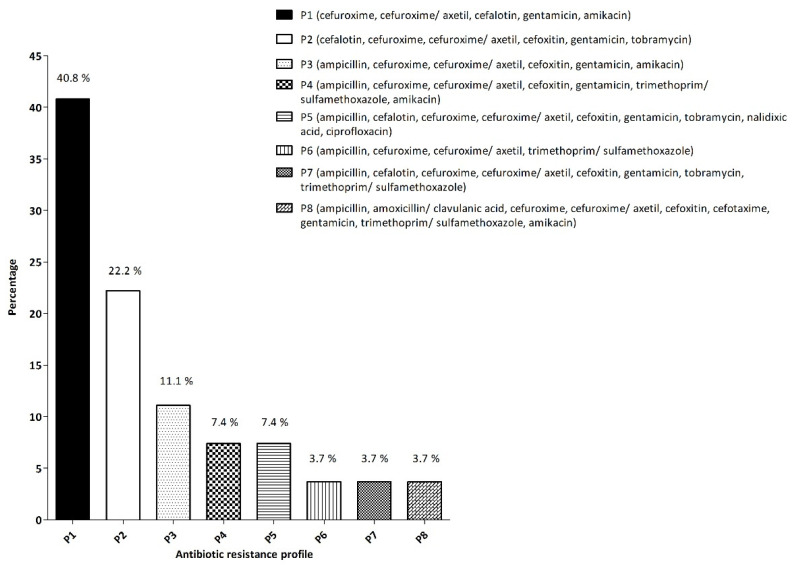
Antibiotic resistance profiles obtained from the 27 isolates.

All 27 isolated strains presented MDR, as all them were resistant to at least 4 antimicrobials. Furthermore, 11 were resistant to 5 antimicrobials, and 3 to 9 of the drugs tested. None of the isolates were sensitive to all of the antimicrobials tested. All the isolates were resistant to cefuroxime and cefuroxime/axetil, a second generation cephalosporin. Similar to previous presentations, all 27 strains can be classified into 8 different profiles attending to their antimicrobial resistance characteristics. This would be: P1 (cefuroxime, cefuroxime/axetil, cefalotin, gentamicin and amikacin); P2 (cefalotin, cefuroxime, cefuroxime/axetil, cefoxitin, gentamicin and tobramycin); P3 (ampicillin, cefuroxime, cefuroxime/axetil, cefoxitin, gentamicin and amikacin); P4 (ampicillin, cefuroxime, cefuroxime/axetil, cefoxitin, gentamicin, trimethoprim/sulfamethoxazole and amikacin); P5 (ampicillin, cefalotin, cefuroxime, cefuroxime/axetil, cefoxitin, gentamicin, tobramycin, nalidixic acid and ciprofloxacin); P6 (ampicillin, cefuroxime, cefuroxime/axetil and trimethoprim/sulfamethoxazole); P7 (ampicillin, cefalotin, cefuroxime, cefuroxime/axetil, cefoxitin, gentamicin, tobramycin and trimethoprim/sulfamethoxazole) and P8 (ampicillin, amoxicillin/clavulanic acid, cefuroxime, cefuroxime/axetil, cefoxitin, cefotaxime, gentamicin, trimethoprim/sulfamethoxazole and amikacin). Most of the isolates tested belonged to P1, as depicted in Figure 3 and summarized in Table 4.

Previous studies, conducted by Martinez-Urtaza et al., on *Salmonella* isolated in the same area of study, indicated a higher incidence of strains resistant to certain antibiotics, such as ceftazidime, or in agreement with the findings reported in the present work, such as the incidence in amoxicillin/clavulanic acid resistance [49]. However, it is worth commenting that further serotyping information would be needed for a better comparison, as the cited study only focused on *S. senftenberg*, and variability in the resistance profiles can exist linked to specific serovars. Martinez-Urtaza et al. also evaluated the resistance profile of *S. typhimurium* isolates from bivalve mollusks, from 1998 to 2002 [16]. They found that 69.6% of isolates were sensitive to all the antimicrobials tested while, in the present study, the 4 *S.* Typhimurium presented multidrug resistance. These results follow the trend of the last few years, in which an increase in resistance has been observed in non-typhoidal *Salmonella* [50]. For example, Giacometti et al. observed an increased trend in the antimicrobial resistance profile of *S. typhimurium* strains isolated from bivalve mollusks collected from Ferrara, Italy, between 2001 and 2017 [51]. In Spain, the same type of trend was observed in strains of *S. enterica* isolated from chicken samples, in which the average number of resistances increased from 3.98% in 1993 to 5.00% in 2006, and an increased incidence in the resistance to cephalothin, enrofloxacin and tetracycline was also observed [52].

It is worth noting that resistance among these isolates was not limited to one type of antibiotic. As it can be observed in pattern 8 of Figure 3, three isolates were resistant to β-lactams (ampicillin, cefalotin, cefuroxime, cefuroxime/axetil and cefoxitin), aminoglycosides (gentamicin and tobramycin) and quinolones (nalidixic acid and ciprofloxacin), covering three different classes of antibiotics. It is also important to note that isolates within this resistance profile, namely AMC 270 and AMC 281, belonged to the virulence profiles 6 (*invA*/*sopB*) and 7 (*invA*/*spvC*/*hilA*/*sopB*) respectively, thus presenting additional virulence factors, and possibly posing an added risk.

Giacometti et al. reported low resistance to third generation cephalosporins and quinolones on isolates of *S. typhimurium*. These finding were in line to those reported in the present study. Contrary to their results, 96.3% of the isolates, included in the current study, were resistant to gentamicin, while Giacometti et al. only observed 1.9% [51]. These results also differ from those observed by Zahli et al., who did not report any isolate resistant to gentamicin from mollusks from the North Coast of Morocco [28]. Additionally, Martinez-Urtaza et al. found only one isolate of *S. typhimurium* that was resistant to gentamicin, among the ones studied from marine environments between 1998 and 2002 [16]. Interestingly, the high resistance to gentamicin has not been observed in *Salmonella* isolated from other points of the food chain in Galicia. Lamas et al. found that all the *Salmonella* isolated from poultry farms from 2011–2015 were sensitive to gentamicin [36]. In the EU report on the antimicrobial resistance in zoonotic bacteria in 2015, the data from Spain showed resistance to gentamicin in 3.8% and 6.3% of isolates of *Samonella* from humans and calves, respectively [53].

High resistance to second-generation cephalosporins was observed in this study. In humans, cefuroxime is the fourth most consumed antibiotic per inhabitant in Galicia [54]. This usage could be partly responsible for the high resistance observed in this pathogen; all the isolates were resistant to cefuroxime. This group of antimicrobials are widely used in animal production in Spain, which can cause an environmental pressure that selects bacteria resistant to these antibiotics [55]. Finally, 37.0% of the isolates were resistant to ampicillin. This is one of the most common resistances observed in *Salmonella* [53]. Penicillins are the most widely used group of antibiotics in animal production, which makes these resistances common in foodborne pathogens [55].

Finally, it is also important to highlight the data on intermediate resistances. These were observed for amoxicillin/clavulanic acid, ceftazidime, cefepime, nitrofurantoin and piperacillin-tazobactam. The WHO has classified these substances as critically important antimicrobials (CIAs), with the exception of nitrofurantoin [56]. Of particular interest is the antimicrobial profile of *Salmonella* AMC 92, which, in addition to the resistance to nine different antimicrobials, also presented three intermediate resistances to ceftazidime, cefotaxime and piperacillin-tazobactam. All isolates were sensitive to: cefditoren, ceftazidime, cefepime, ertapenem, imipenem, fosfomycin, nitrofurantoin, tigecycline and peperacicline/tazobactam; thus, these antimicrobials still represent a reliable way to treat possible infections associated with these bacteria.

## 4. Conclusions

*S. typhimurim* and *S. senftenberg* are serotypes commonly isolated from mussels and the latter seems to be endemic to the Ria de Arousa. Several strains isolated from mussel samples were positive for more than 1 virulence gene. All isolates were negative for the antimicrobial resistance genes tested, but presented phenotypic resistance to more than 4 antibiotics. Even though the multiresistance seems widespread, the strains under study were still susceptible to another 9 different antibiotics, which can represent the future first line treatment. The authors would like to highlight that depuration procedures are efficient in eliminating *Salmonella* spp. from mussels, and, if detected in this product, it would most likely be the result of a post-depuration contamination rather than the inefficacy of the treatment.

## Figures and Tables

**Table 1:** List of primers for virulence and antimicrobial resistance genes.

Primers	Target Gene	Sequence (5′-3′)	Reference
invA-f	*inv*A	GTG AAA TTA TCG CCA CGT TCG GGC AA	[19]
invA-r		TCA TCG CAC CGT CAA AGG AAC C	
spvC-1	*spv*C	CGG AAA TAC CAT CTA CAA ATA	[18]
spvC-2		CCC AAA CCC ATA CTT ACT CTG	
pefA1	*pef*A	TGT TTC CGG GCT TGT GCT	[20]
pefA2		CAG GGC ATT TGC TGA TTC TTC C	
hilA DS	*hil*A	CGG AAG CTT ATT TGC GCC ATG CTG AGG TAG	[21]
hilA US		GCA TGG ATC CCC GCC GGC GAG ATT GTG	
sopB PRSB1	*sop*B	CCA CCG TTC TGG GTA AAC AAG AC	[22]
sopB PRSB2		AGG ATT GAG CTC CTC TGG CGA T	
armA-f	*arm*A	TAT GGG GGT CTT ACT ATT CTG CCTAT	[23]
armA-r		TCT TCC ATT CCC TTC TCC TTT	
rmtA-f	*rmt*A	CTA GCG TCC ATC CTT TCC TC	
rmtA-r		TTT GCT TCC ATG CCC TTG CC	
rmtB-f	*rmt*B	TCA ACG ATG CCC TCA CCT C	
rmtB-r		GCA GGG CAA AGG TAA AAT CC	
rmtC-f	*rmt*C	GCC AAA GTA CTC ACA AGT GG	
rmtC-r		CTC AGA TCT GAC CCA AC AAG	
rmtD-f	*rmt*D	CTG TTT GAA GCC AGC GGA ACG C	
rmtD-r		GCG CCT CCA TCC ATT CGG AAT AG	
npmA-f	*npm*A	CTC AAA GGA ACA AAG ACG G	
npmA-r		GAA ACA TGG CCA GAA ACT C	
CTX-M-15-F1	*bla*_CTX-M-15_	ATA AAA CCG GCA GCG GTG	[24]
CTX-M-15-F2		GAA TTT TGA CGA TCG GGG	

**Table 2:** Thermal profiles set for each genetic target.

Gene	Hot-Start	Cycles	Denaturalization	Hybridization	Extension	Final Extension	Fragment Size (bp)
*inv*A	94 °C/5 min	30	93 °C/1 min	42 °C/1 min	72 °C/2 min	72 °C/4 min	284
*spv*C							669
*pef*A	94 °C/5 min	25	94 °C/55 s	55 °C/55 s	72 °C/55 s	72 °C/10 min	700
*hil*A	94 °C/3 min	30	94 °C/1 min	65 °C/1 min	72 °C/1 min	72 °C/10 min	854
*sop*B	94 °C/5 min	30	94 °C/1 min	55 °C/1 min	72 °C/2 min	72 °C/10 min	1348
*arm*A	94 °C/5 min	30	94 °C/1 min	55 °C/1 min	72 °C/2 min	72 °C/10 min	514
*rmt*A							635
*rmt*B							459
*rmt*C							752
*rmt*D							375
*npm*A							641
*bla*_CTX-M-15_							483

**Table 3:** *Salmonella* strains isolated in the present study.

Code	Strain	Year of Isolation	Origin
AMC 28	*S. montevideo* *	2012	Ria de Arousa
AMC 90	*S. rissen* *	2014	Ria de Arousa
AMC 92	*Salmonella* spp.	2014	Ria de Arousa
AMC 93	*Salmonella* spp.	2014	Ria de Arousa
AMC 200	*S. wentworth* *	2014	Ria de Arousa
AMC 238	*S. typhimurium* *	2015	Ria de Arousa
AMC 239	*S. rissen* *	2015	Ria de Arousa
AMC 240	*S. rissen* *	2015	Ria de Arousa
AMC 256	*Salmonella* spp.	2015	Ria de Arousa
AMC 257	*S. offa* *	2015	Ria de Arousa
AMC 265	*S. montevideo* *	2015	Ria de Arousa
AMC 266	*S. senftenberg* *	2015	Ria de Arousa
AMC 267	*S. senftenberg* *	2015	Ria de Arousa
AMC 268	*S. typhimurium* *	2015	Ria de Vigo
AMC 270	*S. agona* *	2015	Ria de Vigo
AMC 281	*Salmonella* spp.	2015	Ria de Arousa
AMC 287	*Salmonella* spp.	2015	Ria de Arousa
AMC 288	*Salmonella* spp.	2015	Ria de Arousa
AMC 289	*S. senftenberg* *	2015	Ria de Arousa
AMC 290	*Salmonella* spp.	2015	Ria de Arousa
AMC 291	*S. typhimurium* *	2015	Ria de Arousa
AMC 294	*S. typhimurium* *	2015	Ria de Arousa
AMC 299	*S. typhimurium* *	2015	Ria de Arousa
AMC 300	*Salmonella* spp.	2015	Ria de Vigo
AMC 301	*S. bredeney* *	2015	Ria de Arousa
AMC 303	*Salmonella* spp.	2016	Ria de Vigo
AMC 327	*S. liverpool* *	2016	Ria de Vigo

* Serotype information obtained from Lozano-Leon et al. [27] in this reference additional molecular information may be obtained for the designated strains.

**Table 4:** Summary of the antibiotic resistance and virulence genes for each of the strains isolated.

		Antibiotics																							Virulence Genes											
Strain	AMC Code	Area	AMP	AMC	CEF	CXM	CXM/axetil	FOX	CDN	CTX	CAZ	FEP	ETP	IPM	GEN	TOB	NAL	CIP	FOF	NIT	SXT	AMK	TGC	TZP	*invA*	*spvC*	*pefA*	*hilA*	*sopB*	*armA*	*rmtA*	*rmtB*	*rmtC*	*rmtD*	*npmA*	*bla*_CTX-M-15_
*S. montevideo* *	28	A	S	S	**R**	**R**	**R**	**R**	S	S	S	S	S	S	**R**	**R**	S	S	S	S	S	N	N	N	**+**											
*S. rissen* *	90	A	**R**	S	**R**	**R**	**R**	**R**	S	S	S	S	S	S	**R**	**R**	S	S	S	S	R	N	N	N	**+**											
*Salmonella* spp.	92	A	**R**	**R**	N	**R**	**R**	**R**	N	**R**	I	I	S	S	**R**	N	S	S	N	N	**R**	**R**	S	I	**+**											
*Salmonella* spp.	93	A	**R**	I	N	**R**	**R**	**R**	N	S	S	S	S	S	**R**	N	S	S	N	N	**R**	**R**	S	S	**+**											
*S. wentworth* *	200	A	S	S	**R**	**R**	**R**	**R**	S	S	S	S	S	S	**R**	**R**	S	S	S	S	S	N	N	N	**+**											
*S. typhimurium* *	238	A	**R**	S	N	**R**	**R**	**R**	N	S	S	S	S	S	**R**	N	S	S	N	N	S	**R**	S	S	**+**											
*S. rissen* *	239	A	**R**	S	N	**R**	**R**	**R**	N	S	S	S	S	S	**R**	N	S	S	N	N	S	**R**	S	S	**+**											
*S. rissen* *	240	A	**R**	S	N	**R**	**R**	**R**	N	S	S	S	S	S	**R**	N	S	S	N	N	S	**R**	S	S	**+**											
*Salmonella* spp.	256	A	**R**	S	N	**R**	**R**	**R**	N	S	S	S	S	S	**R**	N	S	S	N	N	R	**R**	S	S	**+**			**+**	**+**							
*S. offa* *	257	A	S	S	N	**R**	**R**	**R**	N	S	S	S	S	S	**R**	N	S	S	N	N	S	**R**	S	S	**+**											
*S. montevideo* *	265	A	S	S	N	**R**	**R**	**R**	N	S	S	S	S	S	**R**	N	S	S	N	N	S	**R**	S	S	**+**											
*S. senftenberg* *	266	A	S	S	**R**	**R**	**R**	**R**	S	S	S	S	S	S	**R**	**R**	S	S	S	S	S	N	N	N	**+**			**+**	**+**							
*S. senftenberg* *	267	A	S	S	**R**	**R**	**R**	**R**	S	S	S	S	S	S	**R**	**R**	S	S	S	S	S	N	N	N	**+**	**+**		**+**								
*S. typhimurium* *	268	V	S	S	**R**	**R**	**R**	**R**	S	S	S	S	S	S	**R**	**R**	S	S	S	S	S	N	N	N	**+**	**+**	**+**	**+**	**+**							
*S. agona* *	270	V	**R**	S	**R**	**R**	**R**	**R**	S	S	S	S	S	S	**R**	**R**	**R**	**R**	S	I	S	N	N	N	**+**				**+**							
*Salmonella* spp.	281	A	**R**	S	**R**	**R**	**R**	**R**	S	S	S	S	S	S	**R**	**R**	**R**	**R**	S	I	S	N	N	N	**+**	**+**		**+**	**+**							
*Salmonella* spp.	287	A	S	S	N	**R**	**R**	**R**	N	S	S	S	S	S	**R**	N	S	S	N	N	S	**R**	S	S	**+**	**+**										
*Salmonella* spp.	288	A	S	S	N	**R**	**R**	**R**	N	S	S	S	S	S	**R**	N	S	S	N	N	S	**R**	S	S	**+**											
*S. senftenberg* *	289	A	S	S	N	**R**	**R**	**R**	N	S	S	S	S	S	**R**	N	S	S	N	N	S	**R**	S	S	**+**	**+**										
*Salmonella* spp.	290	A	S	S	N	**R**	**R**	**R**	N	S	S	S	S	S	**R**	N	S	S	N	N	S	**R**	S	S	**+**			**+**	**+**							
*S. typhimurium* *	291	V	S	S	**R**	**R**	**R**	**R**	S	S	S	S	S	S	**R**	**R**	S	S	S	S	S	N	N	N	**+**	**+**										
*S. typhimurium* *	294	A	**R**	I	N	**R**	**R**	S	N	S	S	S	S	S	**S**	N	S	S	N	N	**R**	S	S	I	**+**											
*S. typhimurium* *	299	A	S	S	N	**R**	**R**	**R**	N	S	S	S	S	S	**R**	N	S	S	N	N	S	**R**	S	S	**+**											
*Salmonella* spp.	300	V	S	S	N	**R**	**R**	**R**	N	S	S	S	S	S	**R**	N	S	S	N	N	S	**R**	S	S	**+**											
*S. bredeney* *	301	A	S	S	N	**R**	**R**	**R**	N	S	S	S	S	S	**R**	N	S	S	N	N	S	**R**	S	S	**+**											
*Salmonella* spp.	303	V	S	S	N	**R**	**R**	**R**	N	S	S	S	S	S	**R**	N	S	S	N	N	S	**R**	S	S	**+**			**+**	**+**							
*S. liverpool* *	327	V	S	S	N	**R**	**R**	**R**	N	S	S	S	S	S	**R**	N	S	S	N	N	S	**R**	S	S	**+**											

“S”: Sensitive, “R”: Resistant, “I”: Intermediate and “N”: Not tested. “+”: positive result for the corresponding gene. * Serotype information obtained from Lozano-Leon et al. [27]. In this reference, additional molecular information can be obtained for the designated strains AMP: ampicillin; AMC: Amoxicillin/clavulanic acid; CEF: cephalothin; CXM: cefuroxime; FOX: cefoxitin; CDN: cefditoren; CTX: cefotaxime; CAZ: ceftazidime; FEP: cefepime; ETP: ertapenem; IPM: imipenem; GEN: gentamicin; TOB: tobramycin; NAL: nalidixic acid; CIP: ciprofloxacin; FOF: fosfomycin; NIT: nitrofurantoin; SXT: trimethoprim-sulfamethoxazole; AMK: amikacin; TGC: tigecycline and TZP: piperacillin-tazobactam.
